# Transesophageal echocardiographic assessment of right ventricular function in heart transplantation: Preoperative, intraoperative, and postoperative considerations

**DOI:** 10.1016/j.jhlto.2026.100544

**Published:** 2026-03-24

**Authors:** Ashley V. Fritz, Courtney Borg, Faiz Saleem, Marc Martinez, Archer K. Martin

**Affiliations:** Department of Anesthesiology, Division of Cardiovascular and Thoracic Anesthesiology, Mayo Clinic, Jacksonville, FL

**Keywords:** Heart transplantation, Right ventricle, Echocardiography, Perioperative management, Right heart failure, Pulmonary hypertension

## Abstract

Right ventricular (RV) dysfunction remains a major contributor of morbidity and mortality after heart transplantation, and yet its perioperative assessment is both technically challenging and frequently inconsistent. Transesophageal echocardiography (TEE) provides for continuous evaluation of RV structure and function and is uniquely positioned to guide real-time clinical decision-making across the course of transplant care. This review presents a clinically focused, multiparametric framework for TEE-based RV assessment in the preoperative, intraoperative, and postoperative realm.

Key quantitative measures including fractional area change, tricuspid annular plane systolic excursion (TAPSE), tricuspid annular systolic velocity (S′), 3-dimensional RV ejection fraction, and RV longitudinal strain provide complementary but incomplete assessments of RV performance. Reliance on any single parameter is insufficient, particularly in the transplanted heart, where altered geometry, loading conditions, and surgical factors significantly influence measurement accuracy. Integration of these indices with qualitative assessment is critical for accurate interpretation.

TEE plays a critical role in defining preoperative RV function and pulmonary vascular burden, guiding intraoperative management, including de-airing and graft assessment, and enabling early detection of complications such as primary graft dysfunction and RV failure. A structured, multifaceted TEE evaluation may reduce diagnostic variability, enhance hemodynamic management, and improve transplant outcomes.

## Background

The treatment of end-stage heart failure with heart transplantation has grown significantly since its inception, with 98,176 adult heart transplants performed from 1992 to 2024.^1^ Although intraoperative transesophageal echocardiography (TEE) is routinely used during heart transplantation, TEE has a Class IIa recommendation for this indication in the American College of Cardiology/American Heart Association/American Society of Echocardiography guideline for the clinical application of echocardiography.^2^ TEE is used prior to cardiopulmonary bypass (CPB) to evaluate biventricular function, contractility, volume status, pleural effusions, aortic atherosclerosis, and to identify intracardiac thrombi. It plays a critical role during the transition from CPB, particularly in assessing intracardiac air, right ventricular (RV) function, valvular function, and the absence of intracardiac shunts.^3^ Additionally, TEE guidance helps identify early signs of hemodynamic instability, allowing for prompt intervention to stabilize the patient’s condition.^4^

### Echocardiographic assessment of right ventricular function in orthotopic heart transplant

Primary graft dysfunction (PGD) is a leading cause of early morbidity and mortality after heart transplantation, with RV dysfunction representing a distinct subtype.^5^ The International Society for Heart and Lung Transplantation defines PGD-RV as isolated RV failure within 24 hours of transplantation, characterized by elevated right atrial pressure (>15 mmHg), low left atrial or pulmonary capillary wedge pressure (<15 mmHg), and a cardiac index <2.0 L/min/m² with a transpulmonary gradient <15 mmHg or pulmonary artery systolic pressure <50 mmHg or the need for RV assist device placement.^5^ PGD-RV occurs in approximately 10%-30% of transplant recipients and is associated with increased need for inotropic or mechanical circulatory support, longer intensive care unit (ICU) stays, and higher early mortality.^6^ Intraoperative TEE plays a pivotal role in evaluating RV graft function.^3^ Quantitative measures, including fractional area change, tricuspid annular plane systolic excursion, tricuspid annular systolic velocity, and three-dimensional TEE, are integral to a comprehensive functional assessment (Table 1).

**Table 1:** Transesophageal Echocardiographic Parameters for Perioperative Right Ventricular Assessment in Heart Transplantation

Parameters	Clinical utility	Normal reference values	View on Echo	Limitations	References
Fractional area of change (FAC, %)	Best correlation with MRI right ventricular ejection fraction; Incorporates both radial and longitudinal components of right ventricular contraction.	>35%	Planimetry of right ventricular area in mid-esophageal 4-chamber view at end diastole and end systole	Transesophageal echo may measure lower values than TTE due to poor right ventricular apex visualization. Does not account for RVOT	7, 8, 9, 10, 11, 12
Tricuspid annular systolic excursion (TAPSE, mm)	Rapid assessment of right ventricular longitudinal systolic function; Reproducible	>16 mm	Speckle-tracking at lateral tricuspid annulus in mid-esophageal 4-chamber view	May underestimate post-operatively or with annular distortion, angle dependent	8, 9, 10, 11, 12, 13
Tricuspid systolic annular systolic velocity (S′, cm/s)	Assess right ventricular longitudinal systolic function; Less load dependent; easily reproducible	>9.5 cm/s	Tissue doppler at lateral tricuspid annulus in mid-esophageal 4-Chamber view	Angle dependent, may be underestimated by Transesophageal echo	7, 8, 9, 14, 15
3-dimensional right ventricular ejection fraction (%)	Best global right ventricular function assessment, correlates with cardiac MRI. Can be utilized to track recovery.	>45%	3D mid-esophageal-right ventricular focused view	Requires advanced software, expertise, and time-consuming	7, 8, 9, 11, 14, 15, 16, 17
Right ventricular free wall strain (%)	Detects subclinical dysfunction; prognostic value post OHT.	<−20 (more negative)	mid-esophageal 4-chamber (speckle-tracking)	Requires high quality images. Requires advanced software and expertise	7, 8, 9, 10, 12, 14, 15, 18, 19, 20, 21, 22
Qualitative assessment	Visual inspection (right ventricular size, septal motion) Rapid assessment with immediate feedback.	Qualitative	All standard TEE views	Subjective; operator dependent	8, 18, 23, 24, 25, 26

*For heart transplant recipients, combining FAC, TAPSE, and RV free wall strain into a composite score improves outcome prediction.^15^, ^20^

Abbreviations: RVOT, right ventricular outflow tract; TTE, transthoracic echocardiography.

### Fractional area of change

Fractional area change (FAC) is a quantitative measure of RV systolic function applicable to both transthoracic echocardiography (TTE) and TEE. It is calculated from the percent change in RV area between end-diastole and end-systole.^7^

$$\mathrm{FAC}=\frac{{\mathrm{RV}}_{\mathrm{EDA}}-{\mathrm{RV}}_{\mathrm{ESA}}}{{\mathrm{RV}}_{\mathrm{EDA}}}\times 100$$

Normal FAC on TTE is >35%, with <24% predictive of increased need for advanced therapies in the immediate post-heart transplant period and of early PGD. In this context, low FAC correlates with higher inhaled nitric oxide requirements and increased risk of perioperative RV failure, particularly in patients with preexisting pulmonary hypertension or prior mechanical circulatory support.^13^, ^18^, ^27^

Reference ranges for FAC are currently established only for TTE; no validated reference normal values exist for TEE.^28^ While FAC measurements from the two modalities generally agree, TEE values may be slightly lower due to foreshortening and limited visualization of the RV apex.^28^, ^29^ TEE-derived FAC correlates strongly with cardiac MRI-measured RV ejection fraction (gold standard) and with 3-dimensional RV ejection fraction, supporting its use as a surrogate when 3D imaging is not feasible.^28^, ^30^

Acquisition requires an RV-focused mid-esophageal four-chamber view, with precise tracing of the endocardial border at both end-diastole and end-systole, including trabeculations and the moderator band. This can prove particularly difficult near the RV-free wall. Image quality, probe position, gain settings, and border definition critically affect accuracy and contribute to limited interobserver reproducibility and high test–retest variability.^27^, ^30^ FAC does not account for the right ventricular outflow tract (RVOT), which can comprise a substantial portion of RV volume.^31^

While the limitations described above are important to bear in mind, it is also equally important to consider FAC accuracy in relation to other commonly utilized RV function measurements. Compared with tricuspid annular plane systolic excursion (TAPSE) and tricuspid annular systolic velocity (S′), FAC better reflects global RV function because it incorporates both longitudinal and radial components of contraction.^30^, ^32^ Its diagnostic performance is relatively preserved in the transplanted heart, making it a preferred index for routine follow-up despite altered post-transplant contractile patterns.^13^, ^18^, ^29^

### Right ventricular strain

RV strain is commonly used to quantify myocardial deformation by tracking patterns of ultrasound backscatter—referred to as acoustic “speckles”—within the myocardium throughout systole and diastole.^33^ Speckle-tracking echocardiography quantifies the percentage change in myocardial segment length relative to its baseline length across the cardiac cycle, thereby providing an objective measure of RV contractile function.^34^

RV strain is typically obtained using a TTE RV-focused apical 4-chamber view. A comparable TEE assessment can be achieved by modifying the mid-esophageal four-chamber view to create an RV-focused imaging plane analogous to that used in TTE.^8^ Accurate measurement requires complete visualization of the RV free wall throughout the cardiac cycle without dropout or acoustic shadowing, as these artifacts can introduce significant measurement error. Standard reference points are ideally positioned at the lateral tricuspid annulus, septal tricuspid annulus, and RV apex.^8^ When available, dedicated RV speckle-tracking software should be used, as software designed for left ventricular analysis may yield inaccurate RV strain measurements due to differences in ventricular geometry and contraction patterns.^35^

RV strain can be quantified using either RV free-wall longitudinal strain (RV-FWS) or RV global longitudinal strain (RV-GLS). RV-FWS includes measurements from the basal, mid-ventricular, and apical segments of the RV free wall, whereas RV-GLS incorporates all 6 RV segments, including the interventricular septum.^35^ These measurements are not interchangeable, as RV-GLS values are typically approximately 5% points lower in magnitude than RV-FWS values.^35^ Normal RV-FWS values are generally considered more negative than −20% to −25%. Sex-related differences have also been reported, with women demonstrating significantly higher (more negative) strain values compared with men.^36^

During OHT, intraoperative RV strain assessment obtained via TEE has demonstrated high sensitivity for the early detection of graft dysfunction and may provide prognostic information beyond conventional echocardiographic parameters. RV-FWS has been independently associated with incident rejection, the development of cardiac allograft vasculopathy, and mortality among otherwise low-risk transplant recipients.^19^ Nevertheless, no single echocardiographic parameter—including RV strain—can reliably diagnose graft rejection in isolation. The likelihood of rejection increases when abnormalities are present across multiple parameters. One approach to integrating these variables is the myocardial work index, which combines longitudinal strain analysis with pulmonary artery pressure measurements obtained through invasive hemodynamic monitoring to better distinguish physiologic adaptation from subclinical myocardial injury.^37^ This index has demonstrated favorable specificity for detecting rejection related to myocardial damage and may remain preserved even when other longitudinal function indices are abnormal.^37^

Several limitations should be considered when interpreting TEE-derived RV strain measurements during OHT. Positional changes of the transplanted heart and intraoperative hemodynamic fluctuations can significantly affect strain values. Compared with the native heart, the orthotopically transplanted heart is often smaller, more medially positioned, and rotated within the pericardial cavity, which may complicate image acquisition. In addition, strain measurements are sensitive to loading conditions; alterations in preload or afterload may significantly influence measured values.^8^ For example, increased preload may produce apparently improved strain values that do not necessarily correspond to enhanced intrinsic RV systolic function. Accordingly, RV strain should be interpreted in conjunction with other echocardiographic parameters and the broader intraoperative clinical context when evaluating RV performance during transplantation.

### Tricuspid annular plane systolic excursion

TAPSE is a quantitative echocardiographic parameter RV longitudinal systolic function, measured as the displacement of the lateral tricuspid annulus from end-diastole to end-systole in the RV-focused four-chamber view. Although reproducible and widely available, TAPSE evaluates only longitudinal shortening of the RV free wall and does not account for transverse motion or RVOT contraction; therefore, it should be interpreted in conjunction with other indices of RV performance.^14^

In adults, a TAPSE >16 mm is generally considered normal, whereas values <15 mm are associated with adverse postoperative and long-term outcomes after cardiac surgery.^15^ Intraoperatively, TAPSE is typically obtained with TEE from the mid-esophageal 4-chamber view. Angle dependence and altered imaging planes relative to TTE can result in systematically lower values with TEE. Two adaptations have been described: the modified TAPSE (m-TAPSE), defined as the difference in apical-to-lateral annular distance between diastole and systole, and anatomic M-mode TAPSE (AMM-TAPSE), which aligns M-mode along the anatomical axis of annular motion. Both methods demonstrate improved correlation with TTE-derived TAPSE, with normal values exceeding 16 mm.^13^, ^38^

Advantages of intraoperative TAPSE include quantitative, rapid RV function assessment with high reproducibility, as endorsed by the American Society of Echocardiography. However, TAPSE assumes that the motion of a single basal segment reflects global RV function—a premise that may result in overestimation or underestimation of true RV performance.^28^

In OHT recipients, baseline TAPSE is frequently reduced due to diminished longitudinal fiber shortening despite preserved circumferential contraction.^39^ Additionally, changes in load dependence will alter TAPSE results. Mean TAPSE in stable transplant patients approximates 15 ± 4 mm, and persistent values <15 mm have been associated with increased mortality and accelerated cardiac allograft vasculopathy.^39^, ^40^, ^41^ Despite these limitations, TAPSE retains prognostic value in the post-transplant population and remains a useful component of multimodal RV assessment.

### Tricuspid annular systolic velocity (S′)

Tricuspid annular systolic velocity (S′) represents the peak systolic velocity of the lateral tricuspid annulus as assessed by tissue Doppler imaging (TDI) and serves as a robust, yet technically straightforward, surrogate of longitudinal RV systolic function—the dominant contributor to RV ejection dynamics.^42^, ^43^ Measurement is performed by placing a pulsed TDI sample volume at the lateral tricuspid annulus in the four-chamber view, ensuring maximal beam alignment with the direction of annular motion to minimize underestimation.^8^, ^43^ On the TDI tracing, S′ corresponds to the peak positive deflection above baseline during systole, reported in centimeters per second (cm/s).^43^ In adults, normal values are > 9.5 cm/s, with velocities < 9.5 cm/s considered abnormal and indicative of RV systolic dysfunction.^8^, ^42^

S′ is reproducible, correlates well with other global measures of RV systolic performance, and is less load-dependent than many alternative indices.^42^, ^43^ Limitations include its angle dependency and its restriction to the basal RV free wall.^8^ Accuracy may be further reduced in the presence of significant tricuspid regurgitation (TR), annular tethering, or after cardiac surgery—including OHT—where annular motion may be altered.^8^, ^44^

### Three-dimensional transesophageal echocardiography

Three-dimensional transesophageal echocardiography (3D TEE) provides comprehensive volumetric datasets and geometry-independent quantification of RV size and function in the perioperative setting.^45^ Image acquisition may be performed within a single cardiac cycle or over multiple cardiac cycles with electrocardiographic gating and stitching of multiple subvolumes to enhance spatial and temporal resolution. The resulting datasets can be cropped and rotated to generate en face views of cardiac structures—including valves, ventricles, and surgical anastomoses—facilitating detailed anatomical and functional assessment.

Compared with 2-dimensional techniques, 3D TEE enables reproducible measurement of RV end-diastolic and end-systolic volumes as well as RV ejection fraction, demonstrating strong internal validity in perioperative applications.^45^ While the American Society of Echocardiography acknowledges that TEE can provide superior RV imaging compared with TTE in certain contexts, TEE-specific reference values remain poorly defined.^23^ Limitations of 3D TEE include the need for specialized operator training, susceptibility to stitching artifacts in the presence of arrhythmias, acoustic shadowing from surgical equipment, and the absence of universally accepted normal reference values for OHT patients. Despite these challenges, 3D TEE remains a valuable addition to the anesthesiologist’s diagnostic armamentarium when combined with other modalities such as 2D imaging and color/spectral Doppler.^23^, ^45^, ^46^

In cardiac surgical patients, 3D TEE-derived RV volumes and ejection fraction correlate closely with both cardiac MRI and pulmonary artery catheter-derived hemodynamic indices.^9^, ^16^, ^45^, ^47^ Heart transplant recipients often demonstrate reduced RV ejection fraction and RVFWS compared with controls, with values remaining subnormal for up to 12 months post-transplant.^9^, ^10^ Compared with 2D echocardiographic parameters, 3D TEE RV ejection fraction shows stronger correlation with MRI RV ejection fraction, is associated with functional capacity, and provides prognostic insight.^9^, ^15^, ^16^, ^47^, ^48^

Mechanistic studies suggest that OHT recipients rely more heavily on radial free-wall motion for global RV function, underscoring the importance of using 3D-derived measures that integrate both longitudinal and radial components rather than depending solely on TAPSE, S′, or FAC.^17^, ^49^ Collectively, these findings support the role of 3D TEE as a practical and clinically informative modality for perioperative RV quantification in heart transplant recipients.

Current guidelines from the American Society of Echocardiography, the Society of Cardiovascular Anesthesiologists, and the Society of Thoracic Surgeons recommend comprehensive post-implant TEE, including focused RV evaluation and systematic assessment of anastomotic integrity, given the high incidence and clinical consequences of RV dysfunction following OHT.^46^

### Pre-graft implantation right ventricular evaluation (prior to heart transplant graft implantation)

Pre-implantation evaluation of the native RV is a critical component of intraoperative assessment before OHT. After induction of general anesthesia, a comprehensive TEE examination should be conducted and include evaluation of valvular lesions (e.g., tricuspid or pulmonic regurgitation or stenosis), detection of native or device-associated thrombi, assessment of potential anastomotic sites such as the recipient pulmonary artery and left atrial cuff, and screening for extracardiac findings including pleural effusion or ascites.^3^ Estimation of systolic pulmonary artery pressure using peak TR jet velocity combined may assist in characterizing the severity of pulmonary hypertension and anticipating perioperative RV-pulmonary vascular interactions. General anesthesia and the initiation of positive-pressure ventilation can alter RV preload and afterload; a dynamic reassessment of RV size, shape, interventricular septal position, and motion plays a key role in intraoperative management.^50^ The distinction between pressure overload, commonly caused by elevated pulmonary vascular resistance, and volume overload, often secondary to TR or high-flow states, is crucial because RV adaptation and management differ between these conditions. Pressure overload typically results in concentric hypertrophy and septal flattening, while volume overload causes RV dilatation. Anticipating potential hemodynamic instability under these conditions, the anesthesiologist should be prepared to initiate inotropic support, pulmonary vasodilators, inhaled nitric oxide, or early CPB to maintain stability before graft implantation.^51^

### Intraoperative right ventricular evaluation (during heart transplant graft implantation)

Although intraoperative procedures vary among institutions, de-airing is typically performed following completion of the anastomoses. Intraoperative TEE plays a pivotal role in monitoring and guiding de-airing of the heart to minimize the risk of air embolism. TEE provides real-time feedback to the surgical team regarding the adequacy of de-airing and may inform the need for adjunctive maneuvers such as aspiration, use of de-airing ports, or partial aortic clamping.^52^, ^53^

Once an adequate degree of intracardiac air removal has been achieved, the aortic cross-clamps are released, and the reperfusion of the donor organ is initiated. TEE continues to guide the completion of the de-airing process. Upon confirmation of satisfactory de-airing CPB support is discontinued, and a focused post-CPB TEE examination is performed to assess graft function and ensure the absence of residual intracardiac air.^54^

Echocardiographic evaluation of the RV should immediately focus on the new anatomical configuration of the transplanted heart. Particular attention should be directed toward the integrity of the anastomoses. In the bicaval technique, obstruction of the superior or inferior vena cava may occur secondary to a stenosis at a narrow anastomotic site.^54^ Likewise, narrowing at the pulmonary artery anastomosis may result in RV dysfunction secondary to increased afterload pressures. Timely and systematic echocardiographic assessment of the anastomoses allows for prompt identification and surgical correction of such complications. Echocardiographers should meticulously evaluate anastomotic patency throughout the perioperative period, with particular attention to redundant tissue or sutures that may compromise flow. Similarly, obstruction of the vena cava may occur secondary to thrombus formation, leading to inflow obstruction with concordant rapid clinical decompensation as noted in prior case reports. In both scenarios, Intraoperative TEE is instrumental in isolating the source of obstruction and guiding in corrective surgical intervention.^55^, ^56^, ^57^, ^58^ A focused post-CPB echocardiographic examination should incorporate both qualitative and quantitative assessments. The integration of these modalities is fundamental in cardiac surgery, as experienced echocardiographers can reliably identify cases in which RV function is clearly preserved or overtly impaired. In contrast, borderline RV performance necessitates confirmatory quantitative evaluation using objective parameters.^59^

Key qualitative aspects in the post-transplant assessment include the identification of volume and pressure overload states. Tricuspid or pulmonary valve regurgitation may precipitate volume overload, manifesting as diastolic leftward septal shift and RV dilation. Conversely, pressure overload due to pulmonary valve pathology or anastomotic stenosis at the pulmonary artery can lead to systolic septal flattening or leftward deviation, resulting in biventricular dysfunction.^54^

Commonly utilized quantitative indices include TAPSE, S′, FAC, and RVFWS.^7^ RV dimensions should be assessed at 3 levels—length, base, and mid-level—with normal values ranging from 5.9–8.3 cm, 2.5–4.1 cm, and 1.9–3.5 cm, respectively.^54^ Basal diameters greater than 4.1 cm or mid-level diameters exceeding 3.5 cm suggest RV dilation, though reference ranges should be interpreted in the context of body surface area, sex, and height. RV systolic function should be assessed using multiple parameters; TAPSE <17 mm, FAC <45%, and S′ <9.5 cm/s are indicative of systolic dysfunction. Increased afterload may precipitate intraventricular septal flattening and leftward shift, which can be quantified using the eccentricity index obtained from the trans-gastric window.^54^, ^59^ RV ejection fraction and RVFWS may provide additional insights into ventricular performance.^54^ A longitudinal RVFWS less negative than –20% may indicate impaired RV systolic function,^60^ whereas FAC demonstrates increased sensitivity but lower specificity.^60^

### Post-operative right ventricular evaluation in the ICU

TEE remains invaluable in the ICU, providing a detailed assessment of RV function and enabling timely differential diagnosis and intervention. It facilitates decision-making regarding advanced support measures, including Extracorporeal Life Support (ECLS), cannulation, cardioversion, and tailored hemodynamic management, thereby enhancing overall stability of care.^61^, ^62^

Several postoperative complications can directly impair RV performance, including pericardial effusion, regional wall motion abnormalities, external compression, and RVOT obstruction. Acute pericardial effusions can be identified in the immediate post-operative period and may evolve into tamponade physiology as localized fluid collections expand. Visceral pericardial thickening secondary to prolonged inflammation can further complicate the assessment of RV wall motion and thickness.^14^, ^18^ Echocardiography remains indispensable for the timely detection of expanding pericardial effusions, hematomas, or external compressive phenomena from mediastinal structures, all of which may precipitate life-threatening RVOT obstruction.^63^

Pulmonary hypertension, elevated pulmonary vascular resistance, and RV dysfunction (due to ischemia–reperfusion injury or valvular abnormalities) are major contributors to RV failure. PGD typically presents within the first 24 hours post-transplantation. Clinically, RV failure most commonly manifests within 24 hours postoperatively but may occur up to 72 hours following transplantation. Often, gradual recovery is seen over the following week; however, a subset of patients requires prolonged inotropic support or mechanical circulatory support during this period.^64^

TR is a frequent finding in heart transplant recipients, with reported incidence varying widely; severe TR has been documented in up to 54% of patients.^65^ Etiologies include pre-existing wall motion abnormalities, papillary muscle injury, RV dilation, pulmonary hypertension, and mechanical trauma from repeated endomyocardial biopsies. Severe TR may in turn exacerbate or precipitate RV dysfunction.^54^

Post-transplant evaluation of RV function should employ a multiparametric approach, as recommended by the European Association of Cardiovascular Imaging.^66^ Reliance on single-parameter assessments is limited by several factors, including alterations in pericardial geometry following transplantation. The donor heart often resides within a pericardial cavity larger than its native configuration, which may modify cardiac motion patterns and influence Doppler-derived measurements.^54^, ^66^ Furthermore, transient systolic and diastolic abnormalities may arise due to myocardial edema, infection, or inflammatory processes.^66^

Concordant abnormalities across multiple echocardiographic parameters may signify early graft rejection.^54^, ^66^ A RV ejection fraction below 45%, TAPSE less than 15 mm, and elevated pulmonary pressures have been associated with graft rejection. Patients with acute rejection commonly demonstrate reductions in tricuspid early (E′) and late (A′) diastolic velocities; however, isolated abnormalities in these parameters are not independently predictive of rejection.^18^

## Conclusion

The comprehensive evaluation of RV through TEE before, during, and after heart transplantation is essential to guiding perioperative management and optimizing patient outcomes. By assessing RV size, function, and pressure dynamics, the anesthesiologist can anticipate and mitigate potential complications such as RV failure, pulmonary hypertension, or TR. This proactive approach, combined with continuous monitoring and timely intervention, ensures that any issues, whether related to ischemia, reperfusion, or graft complications, are addressed promptly. Ultimately, a thorough understanding of the RV's condition throughout the transplant process allows for better decision-making and enhances the likelihood of a smooth post-transplant recovery. Through vigilant and focused RV evaluation, the anesthesiologist can contribute significantly to the patient's hemodynamic stability and the success of the transplant procedure.

## Relevant Disclosures

All of the authors have no financial interests, funding, or acknowledgements to disclose.

## Declaration of Generative AI and AI-Assisted Technologies in the Writing Process

Upon completion of this work, the author(s) used ChatGPT in order to check for grammatical errors and improve readability only. After using this tool/service, the author(s) reviewed and edited the content as needed and take(s) full responsibility for the content of the publication.

## Conflicts of Interest statement

The authors declare that they have no known competing financial interests or personal relationships that could have appeared to influence the work reported in this paper.
